# Gender Preference and its Implications on Reproductive Behavior of Mothers in a Rural Area of West Bengal

**DOI:** 10.4103/0970-0218.45377

**Published:** 2009-01

**Authors:** Indira Dey (Pal), Ramendra Narayan Chaudhuri

**Affiliations:** Department of Community Medicine, North Bengal Medical College, Darjeeling, India; 1 Department of Maternal and Child Health, All India Institute of Hygiene and Public Health, Kolkata, India

A sloka of Atharvaveda says, “The birth of a girl, grant it elsewhere. Here grant a son.” Thousands of years later, this saying stands very true in modern times as well, when, despite the so-called modernity, industrialization, literacy and equality, parents still pray thus.(1)

One of the most significant features of the twentieth century has been the dramatic decline in fertility and explicit preference for smaller families in most parts of East and South Asia; this rather than reducing, has exacerbated the preference for a son, leading to an increased discrimination against daughters.

The most disturbing and alarming aspect of the census report (2001) of India is the sharp fall in the sex ratio of children between the age of 0 and 6 years from 945 in 1991 to 927 in 2001. The adverse child sex ratio in the most prosperous states of the country is in a grim situation. The child sex ratio in states such as Punjab and Haryana, which have the highest per capita income, is as low as 793 and 820, respectively. The unfavorable ratio for the girl child (0–6 years) in the prosperous states of North India could be attributed to gender-selective abortion, thereby interrupting the natural course of reproduction. Therefore, this study was undertaken to assess the attitude of mothers toward their preference for the gender of children and its effect on their reproductive behavior.

## Materials and Methods

We conducted a community-based, cross-sectional, study from June 2003 to May 2004 in the Mollasimla village of Hooghly district of West Bengal; this village is the rural field practice area of the All India Institute of Hygiene and Public Health, Kolkata. The total population of this village is 3176 with 439 households. Approximately 70% of the population comprises Muslims. All the mothers in the reproductive age (15–45 years) having under-five children, residing in Mollasimla village were considered for the study. House-to-house visit was made and 156 mothers were registered for the study; six mothers refused to participate in the study and were left out. Informed consent was obtained from all participants. If the mother was absent during the first visit, two more visits were made to contact them. Data were collected using a predesigned and pretested schedule that included mother's literacy status, socioeconomic condition, number of pregnancies and living children, desire for future pregnancy and gender preference for the child and the use of contraceptive methods. Mother's attitude regarding the number and preference to the gender composition of children and her reproductive behavior was assessed. Data was subjected to Chi-square test and compiled and analyzed for percentages and proportions.

## Results

When the mothers were enquired about the ideal number and gender composition of children a couple should have, a majority (62.8%) of the mothers considered two to be the ideal number of children. None of the mothers were in favor of more than four children. The ideal gender composition of the children was one son and one daughter as considered by 53.8% of the mothers. However, 32.7% of the mothers desired for more sons than daughters, while only 3.8% of the mothers wanted more daughters than sons. A desire for only sons was noted in 11.5% of the mothers compared to 0.6% who wanted only daughters. No response was provided by 2.6% of the mothers [Table 1].

**Table 1:** Distribution of mothers according to their views regarding the ideal number of children in the family and their gender composition. N = 156

Ideal number and gender of children	Number and percentage of mothers	
	
	Number	Percentage
One		
Only one daughter	0	0
Only one son	2	1.2
Two		
Both daughters	1	0.6
One son & one daughter	84	53.8
Both sons	13	8.4
Three		
All daughters	0	0
One son & two daughters	5	3.2
Two sons & one daughter	31	19.9
All sons	3	1.9
Four		
Two sons & two daughters	11	7.1
Three sons & one daughter	2	1.3
No response	4	2.6
Total	156	100

The study revealed [Table 2] that among mothers with one living child, all the mothers with a daughter and no son desired for another child and wanted that child to be a boy. Whereas of the mothers with only one son, 8.7% did not want another child and 43.5% of them desired another son and the remaining (47.8%) wanted a daughter. Among mothers with two or three living children, the proportion of mothers not desiring any more children increased with the increase in the number of living sons. Among mothers with three living children, all the mothers with three daughters desired for a son, but none of the mothers with three sons desired for a daughter although the number of mothers in each subgroup was small. Above parity four, the mothers did not show any desire for any more children irrespective of the gender composition of the children. Therefore, 39.2% of mothers wanted a son in their next pregnancy, while only 8.3% of mothers wanted a daughter in future.

**Table 2:** Distribution of mothers according to their desire for more children and number and gender of living children. N = 156

Number and gender of living children	Number and percentage of mothers			
	
	Don’t want another child	Want more		Total no. (%)
			
		Sons	Daughters	
One				
One daughter only	-	34 (100)	-	34 (100)
One son only	2 (8.7)	10 (43.5)	11 (47.8)	23 (100)
Two				
Both daughters	2 (15.4)	11 (84.6)	-	13 (100)
One son & one daughter	23 (88.5)	3 (11.5)	-	26 (100)
Both sons	12 (92.3)	-	1 (7.7)	13 (100)
Three				
All three daughters	-	2 (100)	-	2 (100)
One son & two daughters	6 (85.7)	1 (14.3)	-	7 (100)
Two sons & one daughter	7 (87.5)	-	1 (12.5)	8 (100)
All three sons	3 (100)	-	-	3 (100)
Four or more	27 (100)	-	-	27 (100)
Total	82 (52.5)	61 (39.2)	13 (8.3)	156 (100)

(Figures in parentheses are in percentages)

All the mothers in the study population were interviewed to assess their contraceptive practice and analyzed according to the number and gender of living children [Table 3]. It was found that, among mothers with a single child, a higher proportion of mothers (39.2%) with only one son were found to use temporary measures of contraception compared to the proportion of mothers (23.5%) with only one daughter using the same contraception. None of the mothers had adopted permanent methods. In case of mothers of two children, only 15.4% of mothers with two daughters had undergone sterilization, while 84.6% of mothers with two sons were using contraceptives. It is evident from Table 3 that at each parity, the percentage of women using any contraceptive increased with the increase in the number of living sons. The difference in the use of contraceptives was found to be statistically significant when the mothers had two children (*P* = 0.00, X_2_ = 23.653).

**Table 3:** Distribution of mothers according to their type of contraceptive practice and number and gender composition of their living children

Number & gender of living children	Type of contraceptive use among mothers			
	
	Use (% of permanent + temporary)	Don’t use (%)	Chi-square	*P* value
One				
One daughter (N = 34)	8 (23.5)	26 (76.5)	1.595	0.21
One son (N = 23)	9 (39.2)	14 (60.8)	-	-
Two				
Both daughters (N = 13)	2 (15.4)	11 (84.6)	-	-
One son & one daughter (N = 26)	23 (88)	3 (11.5)	23.653	0.00
Both sons (N = 13)	11 (85)	2 (15.4)	-	-
Three				
All three daughters (N = 2)	1 (50)	1 (50)	3.524	0.32
One son & two daughters (N = 7)	4 (57)	3 (42.9)	-	-
Two sons & one daughter (N = 11)	7 (88)	1 (12)	-	-
All three sons (N = 3)	3 (100)	-	-	-
Four or more		
Sons < daughters (N = 13)	9 (69)	4 (31)	1.081	0.58
Sons = daughters (N = 6)	4 (67)	2 (33)	-	-
Sons >daughters (N = 8)	7 (88)	1 (12)	-	-

## Discussion

In India, preference for son is very strong and pervasive and has been frequently cited as one of the major obstacles in reducing the national fertility level. Table 1 depicts that 62.8% of mothers considered two to be the ideal number of children. In NFHS 2 study,(2) it was found that 47% of the ever married woman in India considered two to be the ideal number of children and 25% considered three to be the ideal number. It has also been observed that in rural India, 37% of the ever married women wanted more sons than daughters but only 2.1% of them wanted more daughters than sons. In West Bengal, 20.7% of woman wanted more sons, while only 3.4% wanted more daughters. These findings corroborate with the present study.

The present study [Table 2] showed that the desire for the next child to be a boy (39.2%) was greater than the desire for a girl child (8.3%) in the next pregnancy. Similar results were noted in NFHS 2 study;(2) 47% women wanted the next child to be a boy compared to only 11% of those who wanted the next child to be a girl. However, in this national survey, 42% of the mothers who wanted another child said that the gender of the child does not matter.

Malhi and Raina reported a stronger preference for the sons present in urban Himachal Pradesh by.(3) They found that at parity two, not a single woman with two daughters desired to terminate child bearing, while an over whelming majority (86.5%) of women with one son and all women with two sons did not want another child. Even at parity four and above, woman who had no living sons did not want to terminate child bearing. Similar findings was obtained in a study conducted in four urban squatter settlements of Karachi by Hussain *et al*.(4) It was found that additional fertility was predominantly correlated with the gender of the surviving children. Although at the primi parity and second parity, this effect was minimal since women were still actively involved in the process of family building but beyond parity two, additional fertility was more closely associated with the surviving number of sons.

This study also revealed that the percentage of women using a contraceptive method increased with the increase in the number of living sons irrespective of the number of living children [Table 3]. The difference in use of contraceptives with regard to the gender composition of the living children has been found to be statistically significant in mothers with two children (*P* = 0.00, X_2_ = 23.653).

Hussain *et al*.,(4) reported similar observation in the urban squatter settlements of Karachi, where the gender of the surviving children appeared to influence the contraceptive use. Similar findings were also evident in a study by Malhi and Raina(3) carried out in urban Himachal Pradesh. In urban Himachal Pradesh, at each parity, contraceptive acceptance was higher among women who had one or more living sons. A study on the teacher couples in Kerala by Gopalkrishnan(5) revealed that contraceptive acceptance particularly sterilization increased with the number of living children even in this literate population.

**Limitation:** All the mothers of children under five were considered for this study; however, the sample size of 156 mothers may not be adequate for generalization of the results of our study.

Therefore, a disparity between boys and girls is prevalent at the family or community level. Therefore, the mother's preference for a male child should be addressed, and they should be able to recognize the importance of the girl child.
